# Effects and exercise-prescription moderators of Tai Chi on cardiometabolic risk factors in middle-aged and older women: a systematic review and meta-analysis

**DOI:** 10.3389/fcvm.2026.1933311

**Published:** 2026-09-09

**Authors:** Jiani Ma, Wenqi Liu, Jun Li, Dawei Zhou, Tianle Wang

**Affiliations:** 1 School of Physical Education, Shaanxi Normal University, Xi’an, China; 2 School of Art, Beijing Sport University, Beijing, China; 3 China Wushu School, Beijing Sport University, Beijing, China

**Keywords:** blood pressure, cardiometabolic risk, exercise prescription, lipid profile, mind-body exercise, non-pharmacological intervention, older women, Tai Chi

## Abstract

**Introduction:**

Cardiometabolic risk increases substantially in middle-aged and older women, particularly during and after the menopausal transition. Tai Chi is a low-to-moderate intensity mind-body exercise that may offer an accessible non-pharmacological strategy for cardiometabolic risk management. However, women-specific evidence and the role of exercise-prescription characteristics remain unclear. This systematic review and meta-analysis evaluated the effects of Tai Chi on cardiometabolic risk factors in middle-aged and older women and examined whether effects varied by baseline health status, comparator type, and intervention dose.

**Methods:**

PubMed, Web of Science, the Cochrane Library, Embase, and CNKI were searched from inception to September 2025 for randomized controlled trials of structured Tai Chi programmes in women aged 45 years or older. Cardiometabolic outcomes were pooled using random-effects models, with moderator, sensitivity, publication bias, and GRADE analyses conducted where appropriate.

**Results:**

Eighteen randomized controlled trials were included. Tai Chi was associated with significant reductions in systolic blood pressure, diastolic blood pressure, LDL-C, triglycerides, and total cholesterol, whereas no clear effects were observed for HDL-C or fasting blood glucose. However, heterogeneity was substantial for most significant outcomes, and prediction intervals generally crossed the null, indicating that effect magnitude and consistency may vary considerably across future comparable settings. Pulse wave velocity was summarized narratively because only two studies reported this outcome and measurement sites differed. Subgroup analyses suggested that baseline health status, comparator type, and intervention duration might partly explain heterogeneity, although these analyses were exploratory.

**Discussion:**

Tai Chi may improve selected cardiometabolic outcomes in middle-aged and older women, particularly blood pressure and atherogenic lipid markers. However, the certainty and generalizability of these findings remain limited. Larger trials using standardized Tai Chi prescriptions and consistent outcome measures are needed.

**Systematic Review Registration:**

https://www.crd.york.ac.uk/PROSPERO/view/CRD420251146060, PROSPERO, CRD420251146060.

## 1 Introduction

Cardiovascular disease (CVD) remains the leading cause of mortality worldwide, with its burden increasing substantially across midlife and later life (1, 2). In women, cardiometabolic risk often rises markedly during the menopausal transition (3, 4). Menopause-related changes in adiposity distribution, insulin sensitivity, lipid metabolism, endothelial function, and arterial aging may contribute to this unfavorable risk trajectory (3–5). These changes contribute to the clustering of dyslipidaemia, impaired glycaemic control, hypertension, and vascular dysfunction in midlife and older women (3, 4, 6).

Women warrant focused investigation because menopause-related endocrine and metabolic changes are not adequately represented in mixed-sex populations (7, 8). Differences in hormonal status, fat distribution, insulin sensitivity, lipid metabolism, and vascular ageing may influence both baseline cardiometabolic risk and responsiveness to exercise interventions (9, 10). A women-specific synthesis is therefore needed to determine whether exercise can mitigate cardiometabolic deterioration during and after the menopausal transition (3, 7).

Exercise is recommended as an important component of cardiometabolic disease prevention and management (11). Current guidelines advise adults to undertake regular aerobic and muscle-strengthening activities, with additional balance and functional exercises recommended for older adults (11). However, conventional aerobic or resistance exercise may be difficult to sustain for individuals with poor fitness, joint discomfort, impaired balance, or limited access to exercise facilities (12–14).

Tai Chi is a traditional Chinese mind–body exercise that integrates slow rhythmic movements, breathing regulation, weight shifting, postural control, and focused attention (15, 16). It is typically performed at low-to-moderate intensity, requires minimal specialized equipment, and imposes relatively low mechanical stress on the joints (15, 17). Tai Chi may also improve balance, motor function, and fall-related outcomes in older adults (18, 19). Collectively, these characteristics may make Tai Chi a feasible and sustainable exercise option for women in midlife and older age, particularly those who experience barriers to conventional aerobic or resistance exercise (20).

Previous evidence suggests that Tai Chi may improve cardiometabolic health (20–22). Meta-analyses have reported beneficial effects of Tai Chi on glycaemic control in people with type 2 diabetes and on blood pressure in people with hypertension (23, 24). In adults with prehypertension, a 12-month randomised clinical trial reported greater reductions in office systolic blood pressure following Tai Chi than following aerobic exercise, with favorable adherence and safety (20). The effects of Tai Chi may also vary according to intervention duration, weekly frequency, and session duration, suggesting that exercise-prescription characteristics could influence treatment outcomes (25).

However, previous reviews have mainly included mixed-sex populations, focused on a single clinical condition, or examined glycaemic, lipid, and cardiovascular outcomes separately (24–27). Some reviews have also pooled Tai Chi with other traditional Chinese exercises, making it difficult to isolate the specific effects of Tai Chi (28–30). Furthermore, the influence of baseline health status, comparator type, intervention frequency, session duration, and program length remains uncertain (25, 27). Unlike previous meta-analyses, the present review focuses exclusively on middle-aged and older women and evaluates Tai Chi across multiple cardiometabolic outcomes without pooling it with other traditional Chinese exercises. It also examines whether baseline health status, comparator type, and exercise-prescription characteristics may moderate the observed effects. This systematic review and meta-analysis therefore aimed to quantify the effects of Tai Chi on blood pressure, lipid profiles, fasting blood glucose, and vascular function in middle-aged and older women.

## 2 Materials and methods

### 2.1 Study design and registration

This systematic review and meta-analysis was conducted in accordance with the PRISMA 2020 statement (Supplementary material 1) (31). The review protocol was prospectively registered in PROSPERO (CRD420251146060).

### 2.2 Search strategy and selection criteria

PubMed, Web of Science, the Cochrane Library, Embase, and China National Knowledge Infrastructure (CNKI) were searched from database inception to September 2025. The search strategy was developed using the PICOS framework and combined terms related to Tai Chi, middle-aged and older women, comparator conditions, and cardiometabolic outcomes. For CNKI, equivalent Chinese and English search terms were used. The full database-specific search strategies are provided in Supplementary Table 2.

Eligible participants were women aged 45 years or older. Eligible interventions were structured Tai Chi-based programmes. Comparators included no intervention, usual care, health education, aerobic exercise, resistance training, or other non-Tai Chi interventions. Studies were eligible if they reported at least one cardiometabolic outcome, including systolic blood pressure, diastolic blood pressure, high-density lipoprotein cholesterol, low-density lipoprotein cholesterol, triglycerides, total cholesterol, fasting blood glucose, or pulse wave velocity. Only randomized controlled trials were included.

Animal studies, qualitative studies, reviews, protocols, conference abstracts, grey literature, quasi-randomized studies, non-randomized controlled studies, uncontrolled studies, studies without a comparator group, and studies without sufficient data for effect-size calculation were excluded.

### 2.3 Study selection

All records identified through the database searches were imported into Zotero, and duplicates were removed. Two reviewers (MJN and LWQ) independently screened titles and abstracts against the prespecified eligibility criteria. Potentially eligible articles were retrieved and assessed in full text. Discrepancies were resolved through discussion, with a third reviewer (ZDW) consulted when consensus could not be reached. Reasons for exclusion at the full-text stage were recorded, and the study selection process was summarised in a PRISMA flow diagram (31).

### 2.4 Data extraction and transformation

Data were extracted independently by two reviewers (MJN and LJ) using a standardized form, including study and participant characteristics, study design, intervention protocols, comparator conditions, outcome data, and attrition. Baseline and post-intervention means and standard deviations were extracted for each outcome when available. Change scores were extracted directly when available; otherwise, mean changes were calculated by subtracting baseline values from post-intervention values (32). When the corresponding standard deviations were unavailable, they were estimated from the baseline and post-intervention standard deviations using an assumed pre–post correlation coefficient of 0.5, as shown in Equation 1:

$$SD_{\mathrm{diff}}=\sqrt{{\mathrm{SD}}_{\mathrm{pre}}{\;}^{2}+{\mathrm{SD}}_{\mathrm{post}}{\;}^{2}-2r\times SD_{\mathrm{pre}}\times SD_{\mathrm{post}}}$$ (1)

where *r* represents the assumed correlation between baseline and post-intervention measurements. This imputation approach was applied consistently across outcomes for which change-score standard deviations were unavailable.

### 2.5 Bias risk assessment

Two reviewers (MJN and ZDW) independently assessed the risk of bias and methodological quality of the included randomized controlled trials. Risk of bias was assessed using the Cochrane Risk of Bias 2 tool (RoB 2), which evaluates bias arising from the randomisation process, deviations from intended interventions, missing outcome data, outcome measurement, and selection of the reported result (33).

The reporting quality of exercise interventions was assessed using the Tool for the Assessment of Study Quality and Reporting in Exercise (TESTEX) (34). TESTEX evaluates key aspects of exercise trial design, including eligibility criteria, randomisation, allocation concealment, baseline comparability, assessor blinding, and intention-to-treat analysis. It also assesses outcome reporting, between-group comparisons, reporting of point estimates and measures of variability, monitoring of physical activity in control groups, and documentation of exercise intensity and volume. Scores range from 0 to 15, with higher scores indicating better methodological quality and reporting.

Discrepancies were resolved through discussion, with a third reviewer (LJ) consulted when consensus could not be reached.

### 2.6 Outcome

The primary outcomes were systolic blood pressure, diastolic blood pressure, high-density lipoprotein cholesterol, low-density lipoprotein cholesterol, triglycerides, and total cholesterol. Secondary outcomes were fasting blood glucose and pulse wave velocity.

### 2.7 Statistical analysis

Statistical analyses and figure generation were performed using the meta and metafor packages in R (35, 36). Random-effects models were used because clinical and methodological heterogeneity was expected across participant characteristics, baseline health status, intervention protocols, comparator conditions, and study designs. Effect estimates were calculated as standardized mean differences (SMDs) with 95% CIs to allow comparability across outcomes measured on different scales or reported in different units (37). For multi-arm trials contributing more than one eligible comparison to the same meta-analysis, shared intervention or control groups were split proportionally to avoid double-counting participants. Negative values indicated greater reductions in the Tai Chi group than in the comparator group for systolic blood pressure, diastolic blood pressure, low-density lipoprotein cholesterol, triglycerides, total cholesterol, fasting blood glucose, and pulse wave velocity; positive values indicated greater increases in high-density lipoprotein cholesterol.

Between-group differences in change from baseline to post-intervention were used whenever sufficient data were available. Heterogeneity was assessed using Cochran's Q, *τ*^2^, *τ*, and *I*^2^ statistics (38). Prediction intervals were calculated to describe the expected range of effects in future comparable studies (39). *I*^2^ values were interpreted pragmatically as indicating low, moderate, or high heterogeneity, while recognising that such thresholds are approximate and context-dependent.

Prespecified subgroup analyses were conducted to explore categorical sources of heterogeneity on the basis of clinical and methodological considerations. Baseline health status was categorised as healthy participants, participants with cardiometabolic diseases, and participants with arthritis. Comparator type was categorised as no intervention, health education, resistance training, aerobic exercise, or other active comparators. Intervention duration was categorised as more than 8 to 12 weeks, more than 12 to 16 weeks, more than 16 to 20 weeks, and more than 20 weeks. Because several subgroup categories contained only one or a few effect sizes, subgroup analyses were interpreted as exploratory rather than confirmatory.

Meta-regression analyses were conducted to examine continuous moderators, including mean participant age, weekly training frequency, total weekly intervention duration, and sample size (40). These variables were selected to explore potential dose-response patterns and study-size effects. Models were fitted using mixed-effects models with restricted maximum likelihood estimation (40). A two-sided *p* value less than 0.05 was considered statistically significant. Findings from subgroup and meta-regression analyses were interpreted cautiously as exploratory, and *p* values were not used to infer definitive effect modification.

Leave-one-out sensitivity analyses were performed to assess whether pooled estimates were driven by any single study. Publication bias and small-study effects were assessed using funnel plots and Egger's test when at least ten independent effect estimates were available for an outcome, with findings interpreted cautiously because all outcomes were analysed using SMDs (41).

### 2.8 Certainty of evidence

Two reviewers (MJN and LJ) independently assessed the certainty of evidence for each outcome using the Grading of Recommendations Assessment, Development and Evaluation (GRADE) approach. Certainty was rated as high, moderate, low, or very low after considering risk of bias, inconsistency, indirectness, imprecision, and publication bias (42). Discrepancies were resolved through discussion, with a third reviewer (LWQ) consulted when consensus could not be reached.

## 3 Results

### 3.1 Study selection

**Figure 1:**
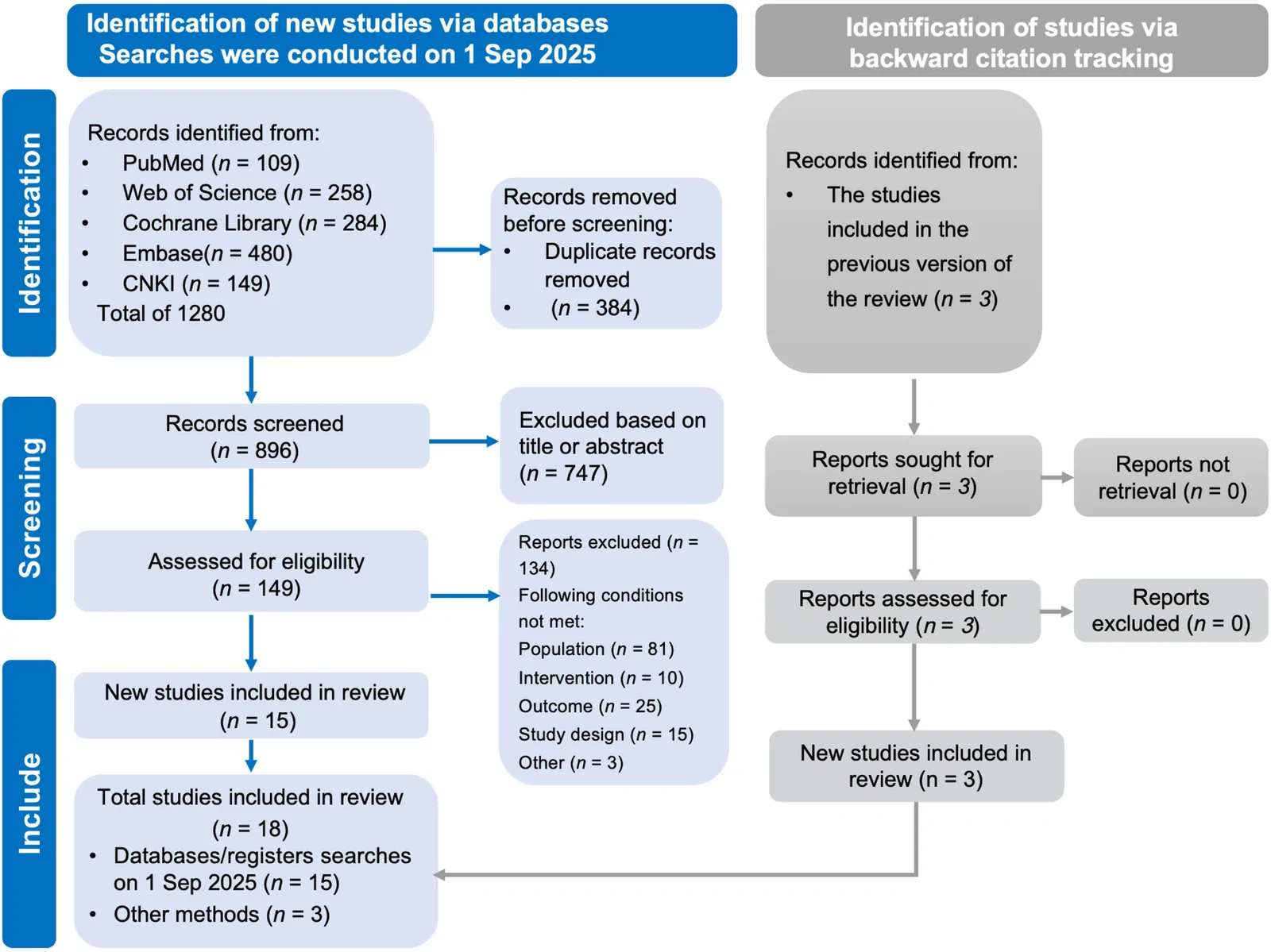
Study selection flow diagram. The template for this figure was adapted from the PRISMA 2020 flow diagram (31), used under the terms of the Creative Commons Attribution 4.0 International License (CC BY 4.0).

The database searches identified 1,280 records: PubMed (*n* = 109), Web of Science (*n* = 258), the Cochrane Library (*n* = 284), Embase (*n* = 480), and CNKI (*n* = 149) (Figure 1). After 384 duplicates were removed, 896 records were screened, and 149 reports were assessed for eligibility. Fifteen studies were included from database searches. Backward citation tracking identified three additional eligible studies, resulting in 18 studies included in the review.

### 3.2 Study characteristics

The review included 18 controlled studies published between 2006 and 2025 (Table 1) (43–60). Studies were conducted in China, Taiwan, South Korea, the United States, and Mexico, with study-level sample sizes ranging from 19 to 154 participants. All participants were middle-aged or older women, including healthy women, postmenopausal women, and women with overweight or obesity, type 2 diabetes, rheumatoid arthritis, knee osteoarthritis, or breast cancer survivors.

**Table 1:** Characteristics of the studies included in the meta-analysis.

Author, Year	Country	Study design	Sample size (*n*)		Age (Year)		Intervention		Health status	Exercise dose	Eligible cardiometabolic outcomes
			EXM	CON	EXM	CON	EXM	CON			
Beebe et al. (43)	United States	Randomized controlled trial	13	13	60.4 ± 6.2	62.6 ± 5.9	24-movement Yang-style Tai Chi + dietary education	Dietary education	Obese older postmenopausal women	45 min/session, 3 sessions/week, 16 weeks	SBP, DBP, HDL-C, LDL-C, TG, TC
Hsu et al. (44)	Taiwan	Randomized controlled trial	56	44	61.75 ± 8.91	63.68 ± 9.63	Yang-style Tai Chi	No intervention	Health	60 min/session, 3 sessions/week, 12 weeks	SBP, DBP
			56	54	61.75 ± 8.91	63.09 ± 8.79	Yang-style Tai Chi	Circuit exercise	Health		
Shin et al. (45)	South Korea	Randomized controlled trial	29	14	64.0 ± 5.4	62.7 ± 5.9	12-form Tai Chi	Health education	Elderly women with rheumatoid arthritis	60 min/session, 1 session/week, 3 months	SBP, DBP, HDL-C, LDL-C, TG, TC, FPG, PWV
Song et al. (46)	South Korea	Randomized controlled trial	29	31	58.6 ± 4.6	61.0 ± 5.6	Health Tai Chi	Health education	Healthy postmenopausal women	60 min/session, 2 sessions/week, 6 months	SBP, HDL-C, LDL-C, TG, TC, FPG
Sprod et al. (47)	United States	Randomized controlled trial	9	10	54.33 ± 3.55	52.70 ± 2.11	Yang-style Tai Chi	Standard support therapy	Female breast cancer survivors	60 min/session, 3 sessions/week, 12 weeks	FPG
Wang et al. (48)	China	Randomized controlled trial	20	20	54.2 ± 4.6	53.7 ± 4.3	Tai Chi, Bafa Wubu	No intervention	Healthy postmenopausal women	60 min/session, 4 sessions/week, 24 weeks	SBP, DBP, HDL-C, LDL-C, TG, TC, PWV
Zhang et al. (49)	China	Randomized controlled trial	10	9	57.4 ± 6.2	57.4 ± 6.2	24-form Tai Chi	No intervention	Women with type 2 diabetes	60 min/session, 5 sessions/week, 14 weeks	SBP, DBP, FPG, TG, TC, HDL-C, LDL-C
Gao et al. (50)	China	Randomized controlled trial	20	20	60.75 ± 3.47	60.23 ± 3.94	Tai Chi Fan	No intervention	Health	30 min/session, 3 sessions/week, 10 weeks	SBP, DBP, TC, TG, HDL-C, LDL-C
Gong et al. (51)	China	Randomized controlled trial	40	40	58.27 ± 2.81	58.34 ± 2.56	Tai Chi Fan	No intervention	Health	60 min/session, 7 sessions/week, 20 weeks	TC, TG, HDL-C, LDL-C
Ma et al. (52)	China	Randomized controlled trial	30	30	60–75	60–75	24-form Tai Chi	No intervention	Health	30–45 min/session, 6 months	SBP, DBP
Sheng et al. (53)	China	Randomized controlled trial	18	18	50–65	50–65	Tui Shou	No intervention	Health	6 months; frequency/session duration NR	SBP, DBP
Song et al. (54)	China	Randomized controlled trial	18	17	63.4 ± 6.67	62.2 ± 7.67	Modified Tai Chi	Health education	Elderly women with knee osteoarthritis	60 min/session, 3 sessions/week, 12 weeks	SBP, DBP
Wang et al. (55)	China	Randomized controlled trial	40	45	56.46 ± 4.51	56.34 ± 3.46	Tai Chi	No intervention	Health	30 min/session, 4 sessions/week, 18 weeks	SBP, DBP
Zhang et al. (56)	China	Randomized controlled trial	23	21	55 ± 4	55 ± 3	Yang-style Tai Chi	Yangko exercise	Health	60 min/session, 3–4 sessions/week, 6 months	SBP, DBP
Zheng et al. (57)	China	Randomized controlled trial	30	30	65.03 ± 6.02	65.75 ± 5.89	Simplified Tai Chi	No intervention	Older postmenopausal women	60 min/session, 5 sessions/week, 12 weeks	SBP, DBP
Duan et al. (58)	China	Randomized controlled trial	18	18	57 ± 1.7	57 ± 1.7	Yang-style Tui Shou and Tai Chi	No intervention	Health	60 min/session, 3–5 sessions/week, 6 months	SBP, DBP
			18	18	57 ± 1.7	57 ± 1.7	Tai Chi	No intervention	Health		
Zhao et al. (59)	China	Randomized controlled trial	60	45	56.65 ± 4.60	55.35 ± 3.56	Simplified Tai Chi	No intervention	Health	30–40 min/session, 3–5 sessions/week, 16 weeks	SBP, DBP
Hernández-Álvarez et al. (60)	Mexico	Randomized controlled trial	19	19	63 ± 4	61 ± 7	8-form Tai Chi	Resistance training	Older women with overweight or obesity	60 min/session, 4 sessions/week, 6 months	TC, TG, HDL-C, FPG

Data are presented as mean ± SD or range where available. EXM, experimental group; CON, control group; SBP, systolic blood pressure; DBP, diastolic blood pressure; HDL-C, high-density lipoprotein cholesterol; LDL-C, low-density lipoprotein cholesterol; TG, triglycerides; TC, total cholesterol; FPG, fasting plasma glucose; PWV, pulse wave velocity; NR, not reported. Exercise dose is presented as session duration, weekly frequency, and intervention duration where available. For multi-arm studies, eligible comparison groups are presented separately.

Tai Chi interventions included Yang-style Tai Chi, 24-form Tai Chi, 12-form Tai Chi, 8-form Tai Chi, simplified or modified Tai Chi, Tai Chi Fan, Tai Chi push-hands, Ba Fa Wu Bu Tai Chi, and tele-training Tai Chi. Intervention duration ranged from 10 weeks to 6 months, with session durations of 30–60 min and weekly frequencies of one to seven sessions. Eligible outcomes included blood pressure, lipid profiles, fasting plasma glucose, and pulse wave velocity.

### 3.3 Risk of bias and methodological quality

Risk-of-bias and methodological quality assessments are shown in Table 2. Six studies were rated as low overall risk of bias, and 12 were rated as having some concerns; no study was judged to be at high overall risk. The main concerns were related to randomisation, deviations from intended interventions, and missing outcome data, whereas outcome measurement and reporting were rated as low risk in all studies. TESTEX scores ranged from 7 to 12 out of 15, with a mean score of 10.8, indicating moderate-to-good methodological quality and reporting of the exercise interventions.

**Table 2:** Risk of bias and methodological quality assessment of the included studies.

Study	Cochrane risk of bias (RoB 2)						TESTEX study quality
	Randomization	Deviation	Missing data	Outcome measurement	Reporting	Overall	
Beebe et al. (43)	Low	Some	Low	Low	Low	Some	9/15
Hsu et al. (44)	Low	Low	Some	Low	Low	Some	11/15
Shin et al. (45)	Some	Some	Some	Low	Low	Some	10/15
Song et al. (46)	Some	Some	Some	Low	Low	Some	11/15
Sprod et al. (47)	Low	Some	Some	Low	Low	Some	7/15
Wang et al. (48)	Low	Low	Low	Low	Low	Low	12/15
Zhang et al. (49)	Some	Some	Some	Low	Low	Some	11/15
Gao et al. (50)	Low	Low	Low	Low	Low	Low	12/15
Gong et al. (51)	Low	Low	Low	Low	Low	Low	12/15
Ma et al. (52)	Some	Some	Low	Low	Low	Some	11/15
Sheng et al. (53)	Some	Low	Low	Low	Low	Some	11/15
Song et al. (54)	Low	Low	Low	Low	Low	Low	9/15
Wang et al. (55)	Low	Low	Low	Low	Low	Low	12/15
Zhang et al. (56)	Some	Low	Some	Low	Low	Some	11/15
Zheng et al. (57)	Low	Low	Low	Low	Low	Low	12/15
Duan et al. (58)	Some	Low	Low	Low	Low	Some	11/15
Zhao et al. (59)	Some	Low	Low	Low	Low	Some	11/15
Hernández-Álvarez et al. (60)	Some	Some	Some	Low	Low	Some	11/15

### 3.4 Pooled main effects of Tai Chi on cardiometabolic outcomes

#### 3.4.1 Effects of Tai Chi on blood pressure

##### 3.4.1.1 Systolic blood pressure

**Figure 2:**
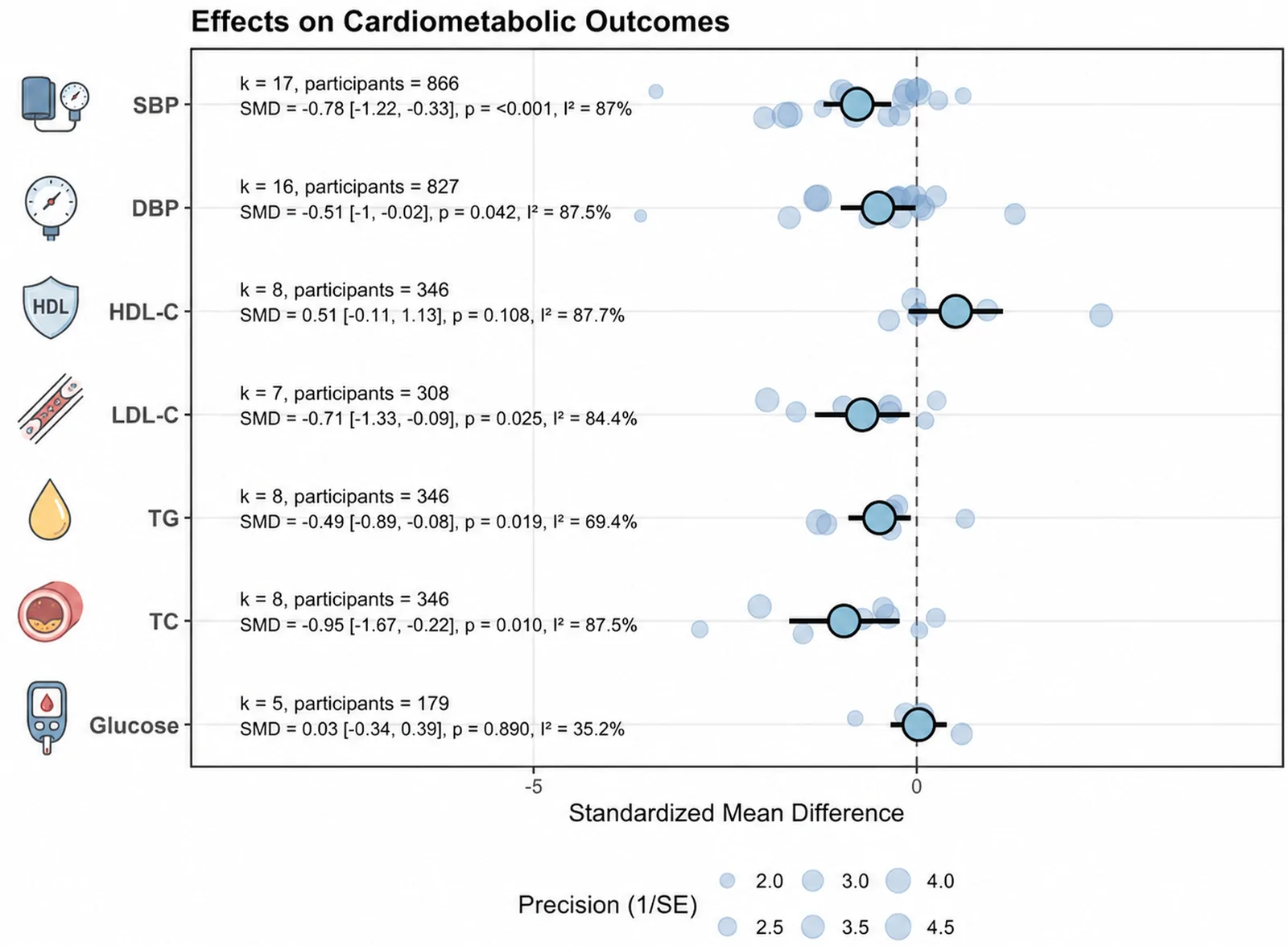
Summary bubble plot of the effects of Tai Chi on cardiometabolic outcomes. Pooled standardized mean differences are shown for blood pressure, lipid profiles, and fasting glucose. Negative values favour Tai Chi for systolic blood pressure, diastolic blood pressure, LDL-C, triglycerides, total cholesterol, and fasting blood glucose, whereas positive values favour Tai Chi for HDL-C. The dashed line indicates the null effect, and circle size reflects study precision. SBP, systolic blood pressure; DBP, diastolic blood pressure; HDL-C, high-density lipoprotein cholesterol; LDL-C, low-density lipoprotein cholesterol; TG, triglycerides; TC, total cholesterol; SMD, standardized mean difference.

Seventeen effect sizes, representing 866 participants, were included. Tai Chi was associated with a statistically significant reduction in systolic blood pressure compared with comparators (SMD = −0.78 [95% CI −1.22 to −0.33], *p* < 0.001; Figure 2; Supplementary Figure 1). However, substantial heterogeneity was observed (*I*^2^ = 87%), and the prediction interval crossed the null (−2.69 to 1.14), indicating that the effect may vary substantially across future comparable studies. Leave-one-out sensitivity analyses showed that the pooled estimate remained statistically significant after omitting each study in turn, suggesting that the average effect was not driven by any single study, although between-study inconsistency remained substantial (Supplementary Figure 2).

Subgroup analyses suggested possible differences in systolic blood pressure effect estimates according to health status, comparator type, and intervention duration (Supplementary Table 3). Larger average reductions were observed in healthy participants, studies using no-intervention controls, and programmes lasting more than 20 weeks. In contrast, effects were not statistically significant in participants with cardiometabolic diseases or arthritis, or in studies using education or active exercise comparators. These patterns may partly reflect differences in comparator intensity, baseline risk, and intervention design.

**Figure 3:**
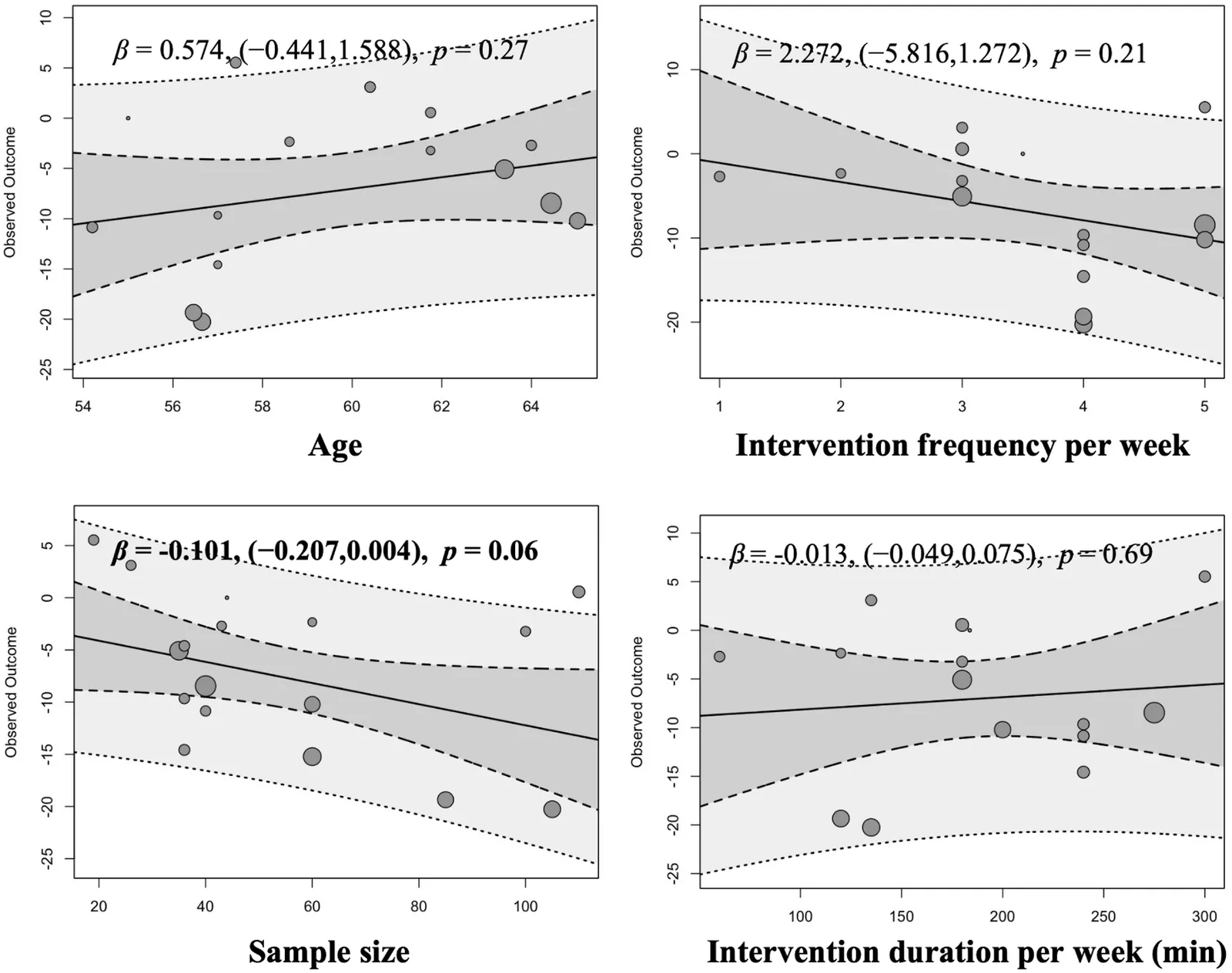
Meta-regression analyses for systolic blood pressure.

Meta-regression analyses showed that age, intervention frequency, and weekly intervention duration were not significant moderators of the effect of Tai Chi on systolic blood pressure (Figure 3). Sample size showed a marginal negative association with the effect estimate (*β* = −0.101 [95% CI −0.207 to 0.004], *p* = 0.06), suggesting that larger studies tended to report greater reductions in systolic blood pressure, although this association did not reach statistical significance.

##### 3.4.1.2 Diastolic blood pressure

Sixteen effect sizes, representing 827 participants, were included. Tai Chi was associated with a statistically significant reduction in diastolic blood pressure compared with comparators (SMD = −0.51 [95% CI −1.00 to −0.02], *p* = 0.042; Figure 2; Supplementary Figure 3). However, substantial heterogeneity was observed (*I*^2^ = 87.5%), and the prediction interval was wide and crossed the null (−2.58 to 1.56), indicating considerable uncertainty about the expected effect in future comparable studies. Leave-one-out sensitivity analyses showed that the effect direction remained negative after omitting each study in turn, but statistical significance was attenuated after excluding several individual studies, suggesting that the diastolic blood pressure finding was less robust than the systolic blood pressure finding (Supplementary Figure 4).

Subgroup analyses suggested possible differences in diastolic blood pressure effect estimates according to intervention duration (Supplementary Table 4), whereas clear subgroup differences were not observed for health status or comparator type. The 16–20 week subgroup showed a statistically significant reduction; however, this estimate was based on only one effect size. Therefore, the apparent duration-related difference should be considered exploratory and should not be interpreted as evidence of a definitive dose-response relationship.

**Figure 4:**
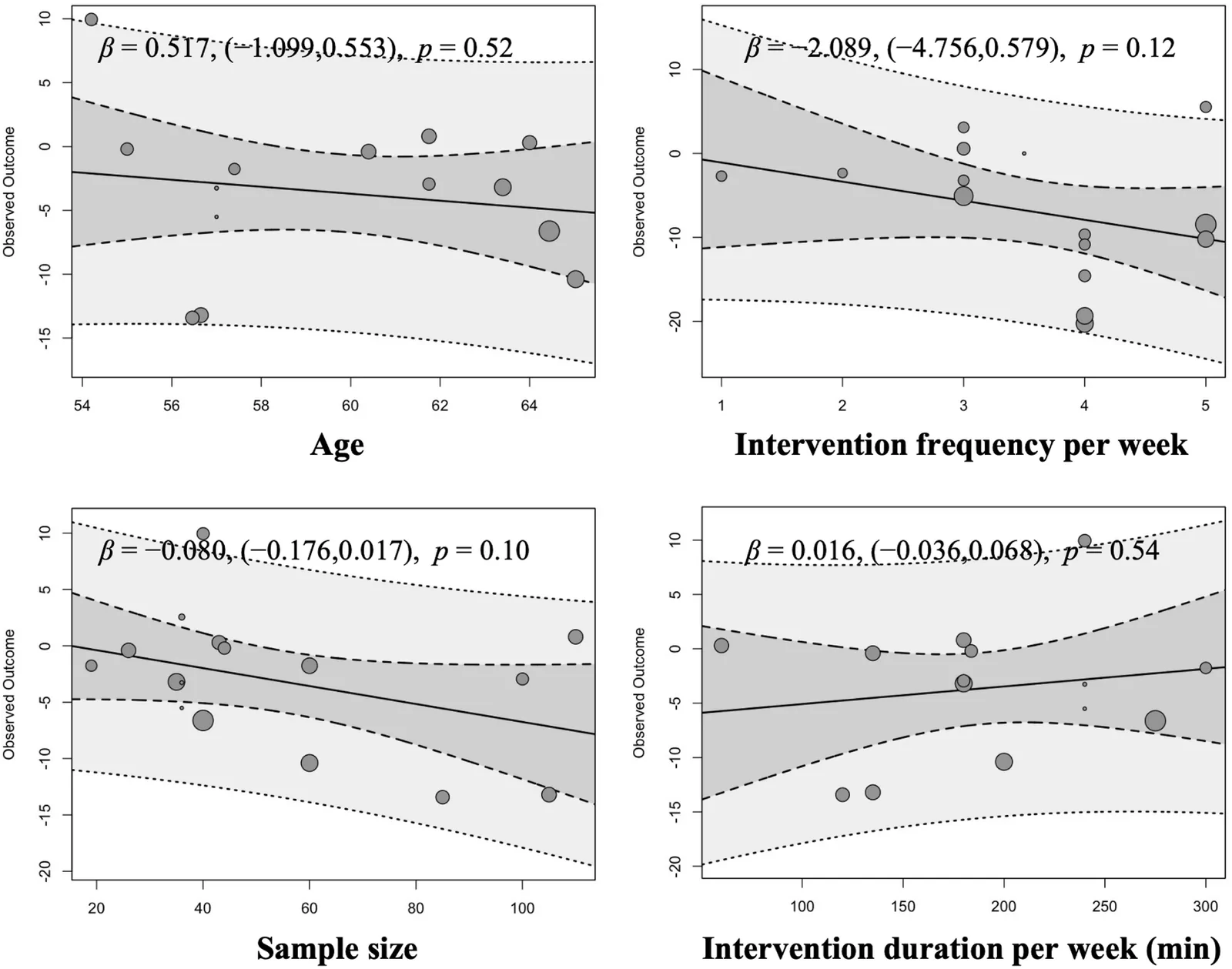
Meta-regression analyses for diastolic blood pressure.

Meta-regression analyses showed that age, intervention frequency, sample size, and weekly intervention duration were not significant moderators of the effect of Tai Chi on diastolic blood pressure (Figure 4). Intervention frequency (*β* = −2.089 [95% CI −4.756 to 0.579], *p* = 0.12) and sample size (*β* = −0.080 [95% CI −0.176 to 0.017], *p* = 0.10) showed non-significant negative trends.

#### 3.4.2 Lipid profiles

##### 3.4.2.1 High-density lipoprotein cholesterol

Eight effect sizes, representing 346 participants, were included. Tai Chi did not significantly increase high-density lipoprotein cholesterol compared with comparators (SMD = 0.51 [95% CI −0.11 to 1.13], *p* = 0.108; Figure 2; Supplementary Figure 5). Substantial heterogeneity was observed (*I*^2^ = 87.7%), with a prediction interval of −1.58 to 2.60. Leave-one-out sensitivity analyses showed that the pooled effect remained non-significant after omitting each study in turn. Heterogeneity decreased markedly after excluding Gong et al., 2009, suggesting that this study contributed substantially to between-study variability (Supplementary Figure 6).

Subgroup analyses suggested possible differences in HDL-C effect estimates according to intervention duration (Supplementary Table 5), while health status and comparator type did not show clear subgroup differences. Statistically significant increases were observed in the 8–12 week and 16–20 week subgroups; however, each of these estimates was based on only one effect size. Given the non-significant overall effect and substantial heterogeneity, these subgroup findings should be regarded as hypothesis-generating only.

##### 3.4.2.2 Low-density lipoprotein cholesterol

Seven effect sizes, representing 308 participants, were included. Tai Chi was associated with a statistically significant reduction in LDL-C compared with comparators (SMD = −0.71 [95% CI −1.33 to −0.09], *p* = 0.025; Figure 2; Supplementary Figure 7). However, substantial heterogeneity was observed (*I*^2^ = 84.4%), and the prediction interval crossed the null (−2.74 to 1.31), indicating that the direction and magnitude of the effect may not be consistent across future comparable studies. Leave-one-out sensitivity analyses showed that the pooled effect remained negative after omitting each study in turn, although statistical significance was attenuated after excluding some individual studies (Supplementary Figure 8). Therefore, this finding should be interpreted as suggestive evidence of a favourable but heterogeneous effect on LDL-C.

Subgroup analyses suggested possible differences in LDL-C effect estimates according to health status, comparator type, and intervention duration (Supplementary Table 6). Larger average reductions were observed in healthy participants, studies using no-intervention controls, and programmes lasting 8–12 weeks, 16–20 weeks, or more than 20 weeks. However, these subgroup patterns should be interpreted cautiously because the number of contributing effect sizes was small and subgroup estimates may be affected by clinical and methodological differences between studies.

##### 3.4.2.3 Triglycerides

Eight effect sizes, representing 346 participants, were included. Tai Chi was associated with a statistically significant reduction in triglycerides compared with comparators (SMD = −0.49 [95% CI −0.89 to −0.08], *p* = 0.019; Figure 2; Supplementary Figure 9). Moderate-to-substantial heterogeneity was observed (*I*^2^ = 69.4%), and the prediction interval crossed the null (−1.73 to 0.76), suggesting uncertainty about the consistency of this effect across future comparable studies. Leave-one-out sensitivity analyses showed that the pooled effect remained negative after omitting each study in turn, although statistical significance was attenuated after excluding some individual studies (Supplementary Figure 10).

Subgroup analyses suggested possible differences in triglyceride effect estimates according to intervention duration (Supplementary Table 7). Differences according to health status and comparator type were less consistent. Larger average reductions were observed in healthy participants, studies using no-intervention controls, and programmes lasting more than 20 weeks. Because subgroup comparisons were based on a limited number of effect sizes, these findings should be interpreted as exploratory rather than confirmatory.

##### 3.4.2.4 Total cholesterol

Eight effect sizes, representing 346 participants, were included. Tai Chi was associated with a statistically significant reduction in total cholesterol compared with comparators (SMD = −0.95 [95% CI −1.67 to −0.22], *p* = 0.010; Figure 2; Supplementary Figure 11). However, substantial heterogeneity was observed (*I*^2^ = 87.5%), and the prediction interval was wide and crossed the null (−3.42 to 1.53), indicating considerable uncertainty about the expected effect in future comparable studies. Leave-one-out sensitivity analyses showed that the pooled estimate remained statistically significant after omitting each study in turn, suggesting that the average effect was not driven by a single study, although the consistency of the effect across settings remains uncertain (Supplementary Figure 12).

Subgroup analyses suggested possible differences in total cholesterol effect estimates according to health status and intervention duration (Supplementary Table 8), whereas comparator type did not show a clear subgroup difference. Larger average reductions were observed in healthy participants, studies using no-intervention controls, and programmes lasting 8–12 weeks, 16–20 weeks, or more than 20 weeks. However, given the substantial heterogeneity and small numbers of effect sizes within several categories, these subgroup results should be interpreted cautiously.

#### 3.4.3 Fasting blood glucose

Five effect sizes, representing 179 participants, were included. Tai Chi did not significantly affect fasting blood glucose compared with comparators (SMD = 0.03 [95% CI −0.34 to 0.39], *p* = 0.890; Figure 2; Supplementary Figure 13). Low-to-moderate heterogeneity was observed (*I*^2^ = 35.2%), with a prediction interval of −0.79 to 0.85. Leave-one-out sensitivity analyses showed that the pooled effect remained non-significant after omitting each study in turn, suggesting no clear evidence of an effect of Tai Chi on fasting blood glucose (Supplementary Figure 14).

#### 3.4.4 Pulse wave velocity

Pulse wave velocity was reported in two studies and was summarised narratively because of differences in measurement sites (Table 3). Shin et al. (45) reported a significant reduction in carotid–ankle pulse wave velocity after 12-form Tai Chi in women with rheumatoid arthritis. Wang et al. (48) reported significant reductions in both left and right carotid–femoral pulse wave velocity after Ba Fa Wu Bu Tai Chi in healthy middle-aged and older women.

**Table 3:** Effect of Tai Chi on pulse wave velocity in middle-aged and older women.

Intervention protocol						
Author, Year	Participants	Type of exercise	Frequency	Session duration	Duration	Main outcomes
Shin et al. (45)	Middle-aged and older women with rheumatoid arthritis	12-form Tai Chi	4 sessions/week	60 min	3 months	Carotid-ankle PWV was significantly reduced (*p* = 0.04*)
Wang et al. (48)	Healthy middle-aged and older women	Ba Fa Wu Bu Tai Chi	4 sessions/week	60 min	24 weeks	The left carotid–femoral PWV was significantly reduced (*p* < 0.01*)
						The right carotid–femoral PWV was significantly reduced (*p* < 0.01*)

Note: *Statistically significant at *p* < 0.05.

### 3.5 Publication bias

**Figure 5:**
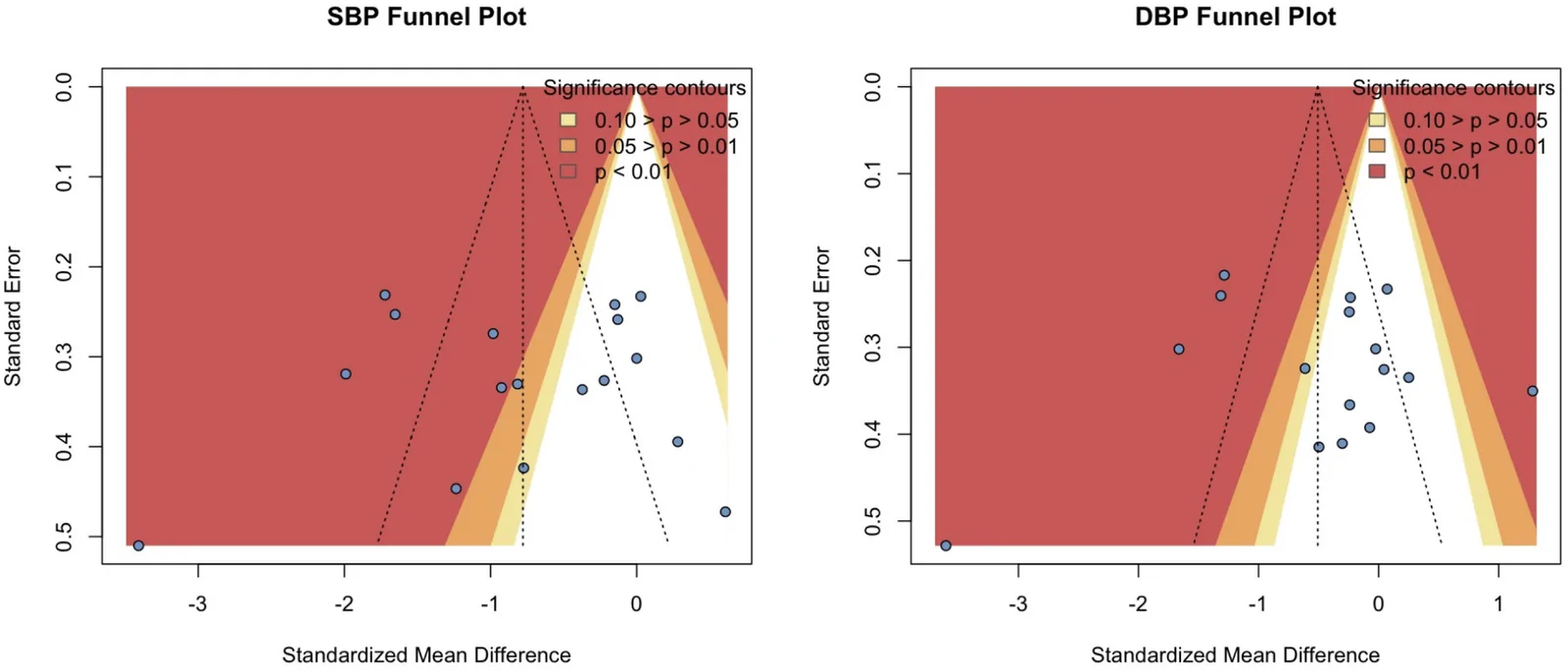
Contour-enhanced funnel plots for blood pressure outcomes. SBP, systolic blood pressure; DBP, diastolic blood pressure.

Visual inspection of the contour-enhanced funnel plots showed no clear evidence of small-study effects (Figure 5). Egger's test did not indicate significant funnel plot asymmetry for systolic blood pressure (*t* = −0.31, *df* = 15, *p* = 0.761) or diastolic blood pressure (*t* = 0.05, *df* = 14, *p* = 0.960).

### 3.6 Certainty of evidence

The certainty of evidence is shown in Table 4. Evidence certainty was rated as moderate for systolic blood pressure, LDL-C, triglycerides, and total cholesterol; low for diastolic blood pressure, HDL-C, and fasting blood glucose; and very low for pulse wave velocity. Downgrading was mainly due to substantial heterogeneity, wide prediction intervals crossing the null for several outcomes, imprecision related to small sample sizes or few effect estimates, and, for pulse wave velocity, the limited number of studies and narrative synthesis. Detailed reasons for downgrading are provided in the GRADE footnotes.

**Table 4:** Certainty of evidence for cardiometabolic outcomes.

Outcome	No of participants (studies)	Certainty assessment					SMD† (95% CI)	Grade
		Risk of bias	Inconsistency	Indirectness	Imprecision	Other		
Taichi group versus control group								
Systolic blood pressure	866 (15 studies; 17 effect sizes)	Not serious	Serious	Not serious	Not serious	None	−0.78 (−1.22 to −0.33)	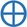 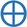 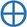 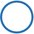 Moderate
Diastolic blood pressure	827 (14 studies; 16 effect sizes)	Not serious	Serious	Not serious	Serious	None	−0.51 (−1.00 to −0.02)	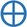 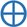 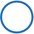 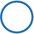 Low
High-density lipoprotein cholesterol	346 (8 studies; 8 effect sizes)	Not serious	Serious	Not serious	Serious	None	0.51 (−0.11 to 1.13)	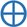 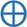 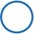 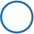 Low
Low-density lipoprotein cholesterol	308 (7 studies; 7 effect sizes)	Not serious	Serious	Not serious	Not serious	None	−0.71 (−1.33 to −0.09)	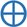 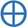 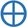 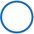 Moderate
Triglycerides	346 (8 studies; 8 effect sizes)	Not serious	Serious	Not serious	Not serious	None	−0.49 (−0.89 to −0.08)	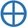 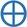 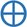 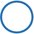 Moderate
Total cholesterol	346 (8 studies; 8 effect sizes)	Not serious	Serious	Not serious	Not serious	None	−0.95 (−1.67 to −0.22)	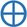 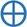 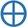 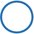 Moderate
Fasting blood glucose	179 (5 studies; 5 effect sizes)	Not serious	Some serious	Not serious	Serious	None	0.03 (−0.34 to 0.39)	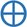 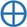 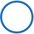 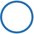 Low
Pulse wave velocity	83 (2 studies)	Not serious	Some serious	Serious	Serious	None	Narrative synthesis	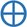 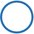 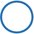 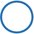 Very Low

Certainty of evidence was assessed according to the Grading of Recommendations Assessment, Development and Evaluation (GRADE) approach.

High certainty indicates that we are very confident in the effect estimate; moderate certainty indicates moderate confidence; low certainty indicates limited confidence; and very low certainty indicates very little confidence in the effect estimate. The number of participants refers to the total number of participants contributing to the pooled effect estimate. For outcomes that were not pooled, findings were summarised narratively.

Downgraded one level for inconsistency because heterogeneity was substantial and/or the prediction interval crossed the null.

Downgraded one level for imprecision because the number of participants or effect estimates was small and/or the confidence interval was wide.

Downgraded for indirectness when studies differed substantially in participant health status, comparator type, or Tai Chi prescription.

SMD, standardized mean difference.

## 4 Discussion

This systematic review and meta-analysis suggests that Tai Chi may improve selected cardiometabolic outcomes in middle-aged and older women, particularly blood pressure and atherogenic lipid markers, although the magnitude of these effects varied across studies. Given that elevated blood pressure and atherogenic lipid levels are major modifiable cardiovascular risk factors, these concurrent improvements may be clinically meaningful as part of a broader risk-management strategy.

These findings are broadly consistent with previous evidence suggesting that Tai Chi may have favourable effects on blood pressure control and lipid metabolism. Unlike many earlier reviews, this study focused specifically on middle-aged and older women and separated Tai Chi from other traditional Chinese exercises. This focus is important because sex, menopausal status, baseline health, and exercise tolerance could influence cardiometabolic responses.

The substantial heterogeneity observed across several outcomes was expected, given differences in participant characteristics, Tai Chi styles, comparator conditions, and intervention duration. Subgroup analyses suggested that effect estimates might differ according to health status, comparator type, and intervention duration, whereas most meta-regression analyses did not identify clear continuous moderators. However, these subgroup findings should be interpreted as exploratory because several categories included only one or a few effect sizes and because subgroup comparisons may be confounded by differences in baseline risk, comparator intensity, and intervention design. Overall, the findings suggest that Tai Chi may be a potentially accessible adjunctive strategy for cardiometabolic risk management, but the evidence remains limited by heterogeneous protocols, small samples for some outcomes, and moderate-to-low certainty of evidence.

### 4.1 Effects on blood pressure and vascular function

The observed reductions in blood pressure are consistent with previous evidence showing blood pressure-lowering effects of Tai Chi in people with hypertension or prehypertension (20, 22, 24, 25). However, the magnitude of the response may vary across clinical populations and intervention protocols. A recent randomized clinical trial also reported greater reductions in office systolic blood pressure after Tai Chi than after aerobic exercise in adults with prehypertension (20). Our review extends this evidence to middle-aged and older women, a population in whom menopause-related vascular ageing, changes in body composition, and increased cardiometabolic risk could modify exercise responses (3–5, 10).

Subgroup analyses suggested possible differences in blood pressure effect estimates according to health status, comparator type, and intervention duration. For systolic blood pressure, larger average reductions were observed in healthy participants and in studies using no-intervention controls. For diastolic blood pressure, apparent differences were mainly observed across intervention-duration categories. These patterns might reflect variation in comparator intensity, baseline blood pressure, clinical context, adherence, or intervention design across studies. However, subgroup results should be interpreted cautiously because several categories included few studies, and some statistically significant subgroup estimates were based on a single effect size.

Pulse wave velocity was reported in only two studies and was not pooled because measurement sites differed. Both studies reported significant reductions after Tai Chi, suggesting potential benefits for arterial stiffness. This finding is biologically plausible given the potential effects of Tai Chi on endothelial function, vascular compliance, and autonomic regulation (48, 61, 62). More trials using standardized pulse wave velocity measurements are needed to confirm these effects. Beyond these vascular outcomes, the favourable changes observed in several atherogenic lipid markers suggest that Tai Chi may also influence metabolic components of cardiovascular risk.

### 4.2 Effects on lipid profiles

Tai Chi was associated with statistically significant average reductions in LDL-C, triglycerides, and total cholesterol, whereas no clear effect was observed for HDL-C. However, the lipid findings should be interpreted cautiously because heterogeneity was substantial for LDL-C and total cholesterol, moderate-to-substantial for triglycerides, and prediction intervals crossed the null. These results suggest that Tai Chi may have favourable effects on atherogenic lipid markers in some contexts, but the consistency and clinical magnitude of these effects remain uncertain (23, 27).

These findings are partly consistent with previous reviews suggesting that Tai Chi can improve lipid metabolism, particularly in people with cardiometabolic risk factors or type 2 diabetes (23, 27). However, the present review focused specifically on middle-aged and older women and excluded other traditional Chinese exercises, which makes the findings more specific to Tai Chi (28–30).

Substantial heterogeneity was observed for most lipid outcomes. Subgroup analyses suggested possible differences in lipid effect estimates according to health status, comparator type, and intervention duration, particularly for LDL-C and total cholesterol. Larger average effects were often observed in healthy participants and in studies using no-intervention controls. These patterns may reflect differences in baseline metabolic status, comparator intensity, adherence, intervention duration, or other study-level characteristics. Because several subgroup estimates were based on a small number of effect sizes, these findings should be considered exploratory and should not be used to define an optimal Tai Chi prescription.

Future trials should use standardized lipid measurements, clearly defined Tai Chi prescriptions, and longer follow-up to determine the consistency and durability of these lipid effects (63, 64).

### 4.3 Effects on fasting blood glucose

Tai Chi did not significantly affect fasting blood glucose in middle-aged and older women. The pooled estimate was close to the null, and leave-one-out sensitivity analyses showed that the finding remained non-significant after removal of individual studies. This result differs from previous evidence suggesting glycaemic benefits of Tai Chi in people with type 2 diabetes (23, 27). One explanation is that the present review included mixed health-status groups, including healthy women and women without elevated baseline glucose. In participants with normal fasting glucose, further reductions might be difficult to detect.

The small number of studies also limits interpretation. Only five effect sizes contributed to the fasting blood glucose analysis, reducing statistical power and increasing uncertainty. Differences in baseline metabolic status, comparator conditions, and intervention dose might also have diluted the pooled effect. Current evidence is therefore insufficient to conclude that Tai Chi improves fasting blood glucose in middle-aged and older women. Taken together, the differing patterns across blood pressure, lipid, and glycaemic outcomes warrant consideration of the physiological pathways through which Tai Chi may exert outcome-specific effects.

### 4.4 Potential mechanisms

Several plausible mechanisms may help explain the observed associations between Tai Chi and improvements in blood pressure and lipid outcomes. Tai Chi combines low-to-moderate intensity movement, breathing regulation, postural control, and focused attention (15, 16). These features might improve autonomic regulation, reduce sympathetic activation, and lower vascular tone, contributing to reductions in blood pressure (65).

Repeated rhythmic movements and dynamic weight shifting during Tai Chi may increase blood flow and vascular shear stress, thereby activating endothelial nitric oxide signalling and promoting vasodilation. These responses may contribute to improvements in endothelial function and arterial stiffness (48, 66, 67). This pathway is consistent with the narrative evidence in this review suggesting reductions in pulse wave velocity.

The lipid effects might be related to Tai Chi-related increases in physical activity, changes in body composition, and improved metabolic regulation. Regular practice could therefore contribute to reductions in LDL-C, triglycerides, and total cholesterol, although the non-significant effect on HDL-C suggests that Tai Chi may not consistently increase protective lipid markers (23, 48, 68).

The absence of a significant effect on fasting blood glucose suggests that glycaemic responses might depend on baseline metabolic status, intervention dose, and comparator condition (69). Future trials should include mechanistic outcomes, such as insulin sensitivity, inflammatory markers, endothelial function, and autonomic function, to clarify how Tai Chi affects cardiometabolic health in middle-aged and older women (70, 71).

### 4.5 Strengths and limitations

This review has several strengths. It focused specifically on middle-aged and older women, a population in whom menopause-related cardiometabolic changes might influence exercise responses. It also separated Tai Chi from other traditional Chinese exercises and examined multiple cardiometabolic outcomes, including blood pressure, lipid profiles, fasting blood glucose, and pulse wave velocity. Subgroup, meta-regression, sensitivity, publication bias, and GRADE analyses were used to assess robustness and certainty of evidence.

This review also has limitations. Substantial heterogeneity was observed for most outcomes, reflecting differences in participant characteristics, Tai Chi style, intervention dose, comparator type, and study design. Some outcomes were based on few studies, especially fasting blood glucose and pulse wave velocity, limiting precision and statistical power. Several subgroup estimates were based on one or only a few effect sizes and should be interpreted cautiously.

Although outcome measurement and reporting were generally rated as low risk, some studies had concerns related to randomisation, deviations from intended interventions, or missing outcome data. Pulse wave velocity was summarised narratively because measurement sites differed across studies. Finally, the included studies varied in intervention reporting, adherence assessment, and follow-up duration, limiting conclusions about the optimal Tai Chi prescription and the durability of effects.

### 4.6 Clinical implications

The findings suggest that Tai Chi may be considered as a feasible adjunctive exercise option for middle-aged and older women, particularly in community-based health promotion and cardiometabolic risk management. Tai Chi requires little equipment, is low impact, and might be suitable for women with limited fitness, joint discomfort, impaired balance, or low exercise tolerance. Exploratory subgroup analyses suggested that generally healthy middle-aged and older women may experience greater average improvements in blood pressure and selected lipid outcomes than women with established cardiometabolic disease or arthritis. However, these observations were based on small numbers of effect sizes and may also reflect differences in baseline risk, comparator intensity, and intervention design; therefore, they should not be used to define a specific target population. Tai Chi should therefore be considered as a complementary lifestyle strategy rather than a replacement for standard clinical care, and current evidence remains insufficient to establish a precise optimal prescription.

## 5 Conclusion

This systematic review and meta-analysis provides women-specific evidence on the effects of Tai Chi on cardiometabolic risk factors in middle-aged and older women. The findings suggest potential benefits for blood pressure and atherogenic lipid markers, whereas effects on HDL-C, fasting blood glucose, and vascular function remain less certain. By focusing exclusively on women and examining potential exercise-prescription moderators, this review addresses an important evidence gap not adequately captured by previous mixed-sex or condition-specific reviews. Tai Chi may therefore represent a feasible complementary exercise strategy for cardiometabolic health promotion in this population. Further adequately powered trials should use standardized Tai Chi protocols that clearly specify the Tai Chi style, exercise intensity, session frequency and duration, and total intervention length, alongside consistent cardiometabolic outcome measures, to improve study comparability and clarify the populations and exercise doses most likely to benefit.

## Data Availability

The original contributions presented in the study are included in the article/Supplementary Material, further inquiries can be directed to the corresponding author/s.
